# Associations of the serotonin transporter gene polymorphism, 5-HTTLPR, and adverse life events with late life depression in the elderly Lithuanian population

**DOI:** 10.1038/s41598-023-40215-4

**Published:** 2023-08-09

**Authors:** Sandrita Simonyte, Ingrida Grabauskyte, Jurate Macijauskiene, Vita Lesauskaite, Vaiva Lesauskaite, Kari Sofie Kvaal, Robert Stewart

**Affiliations:** 1 Institute of Cardiology of Medical Academy, Lithuanian University of Health Sciences, Kaunas, Lithuania; 2 Department of Physics, Mathematics and Biophysics of Medical Academy, Lithuanian University of Health Sciences, Kaunas, Lithuania; 3 Department of Geriatrics of Medical Academy, Lithuanian University of Health Sciences, Kaunas, Lithuania; 4 Faculty of Social and Health Sciences, Inland Norway University of Applied Sciences, Lillehammer, Innlandet Norway; 5 King’s College London (Institute of Psychiatry, Psychology and Neuroscience), London, UK; 6 South London and Maudsley NHS Foundation Trust, London, UK

**Keywords:** Molecular biology, Neurology

## Abstract

Late-life depression (LLD) is a multifactorial disorder, with susceptibility and vulnerability potentially influenced by gene**-**environment interaction. The aim of this study was to investigate whether the *5-HTTLPR* polymorphism is associated with LLD. The sample of 353 participants aged 65 years and over was randomly selected from the list of Kaunas city inhabitants by Residents’ Register Service of Lithuania. Depressive symptoms were ascertained using the EURO-D scale. The List of Threatening Events Questionnaire was used to identify stressful life events that happened over the last 6 months and during lifetime. A *5-HTTLPR* and lifetime stressful events interaction was indicated by higher odds of depression in those with s/s genotype who experienced high stress compared to l/l carriers with low or medium stress, while 5*-HTTLPR* and current stressful events interaction analysis revealed that carriers of either one or two copies of the *s* allele had increased odds of depressive symptoms associated with stress compared to participants with the l/l genotype not exposed to stressful situations. Although no significant direct association was found between the *5-HTTLPR* short allele and depression, our findings demonstrated that lifetime or current stressful life events and their modification by *5-HTTLPR* genotype are risk factors for late-life depression.

## Introduction

Late-life depression (LLD) has been identified as one of the most common mental health disorders affecting up to one third of individuals aged 60 years and older in developed countries^1^. Previous studies indicate that the prevalence of LLD varies among countries and is much higher in Southern and Central Europe than in Western Europe, Scandinavia, or United States^2–5^. Among late-life psychiatric disorders, depression is probably the most frequent cause of emotional suffering and has a significant negative impact on quality of life^6^. LLD is associated with significant cognitive and functional impairment, suicidality, morbidity, and mortality^7,8^. However, risk factors for LLD may differ from the depression found in the younger population^9,10^; for example, older adults are less likely to endorse cognitive-affective symptoms of depression, including dysphoria and worthlessness/guilt, than are younger adults^11^. Alongside physical conditions that accompany depression, multiple social factors may worsen elders’ psychological condition, including loss of family members, friends, work, and social status^12^. Potential protective factors, such as higher education, socioeconomic status, good health, supportive social networks, good cognitive function, engagement in valued activities, and religious involvement may decrease sensitivity to depression in older individuals^13^.

Susceptibility and vulnerability to depression is likely to be influenced by gene-environment interaction (G × E)^14,15^. Specifically, stress-related early life events such as childhood maltreatment or recent negative life events are recognised risk factors for depression onset^16^; however, the response to such life events may be significantly affected by the individual’s genetic profile^17^, although such genetic risk may be more prominent earlier in the lifespan^11^. One of the most investigated genetic polymorphisms related to depression is *5-HTTLPR*, a functional polymorphism in the promoter region of the serotonin transporter (5-HTT) encoding gene (*SLC6A4*). In the brain, the serotonin transporter terminates the action of neurotransmitter serotonin by facilitating its reuptake from the synapse to the presynaptic neuron, thus regulating synaptic serotonin signalling and concentrations^18^. 5-HTT is a main regulator of serotonergic neurotransmission and is a target of the widely used pharmacological treatment such as selective serotonin reuptake inhibitors (SSRIs). The stress-related psychopathology of psychiatric disorders is mainly related to the disturbances in the serotonin system; therefore, interest in *SLC6A4* has persisted^19,20^.

A 44 bp insertion/deletion variable number tandem repeat (VNTR) in the *5-HTTLPR* results in either short (*s*) or long (*l*) alleles^21^. There is evidence that the *s* allele is associated with the lower level of serotonin uptake, lower transcriptional efficiency of the serotonin transporter and poorer response to pharmacological treatment in late-life depression comparing with individuals homozygous for the *l* allele^22–24^. Around 70% of Caucasians have at least one copy of the *s* allele; thus a proposed genetic modifier of the response to environmental factors may have a significant population-level impact^17^. In a well-cited G × E interaction study, Caspi et al.^25^ failed to find any direct association between *5-HTTLPR* and depression but found that childhood maltreatment or other adverse life events in interaction with one or two copies of the *s* allele increased vulnerability to negative psychiatric and behavioural outcomes. Furthermore, this study also demonstrated a dose–response relationship for this interaction, which was stronger in individuals with two s copies than in those with only one. Although further studies^26–29^ and some meta-analyses^30,31^ supported these findings of modification by *5-HTTLPR* genotype and psychiatric disorders, a French community study of elders^32^ and other meta-analyses did not find a significant evidence that the serotonin transporter s/s genotype alone or in interaction with stressful life events was associated with an elevated risk of depression^33–35^. Furthermore, it has been proposed that in younger populations the *s* allele is a sensitive genetic variant for depression, while the *l* allele is a risk factor for mental and physical distress in older groups^36^. Previous studies show that the influence of *5-HTTLPR* can be heterogeneous and highlight possible involvement of other factors and regulatory mechanisms promoting the risk of psychiatric disorders^18,20^. Although some of the previously mentioned studies and meta-analyses have been criticized for including results from studies with incompatible statistical and genetic models, or applying too restrictive inclusion system of studies, the key point is the heterogeneity of the studies^37^. Minimizing heterogeneity factors is necessary to increase the likelihood of detecting a true association between exposure and outcome of interest^35,38,39^.

It should be noted that some of inconsistencies between study findings might be explained by ethnic differences in allele frequency^40^, therefore the evaluation of more homogeneous populations by genetic ancestry and depression-related phenotypes is advantageous for identifying genetic risk factors for this disease^14^. With increasing age there is an accumulation of social risks as stressful life events^11^ that are considered to be among the most important risk factors of LLD; however, how this phenotype is affected by *5-HTTLPR* and/or modified in response to stress in older populations have been rarely studied in previous studies or have reported an inverse association^31^. So far, no similar studies have been conducted in the Lithuanian population. Thus, to address these knowledge gaps, we investigated the *5-HTTLPR* polymorphism for late-life depressive symptoms in response to recent and lifetime stressful life events in an elderly Lithuanian population.

## Results

Descriptive data of Kaunas Healthy Ageing Study are presented in Table 1. There were twice as many females (67.1%) as males. Nearly half of the responders were married or had stable partners (43.4%) and the majority of the remainder were widowed (41.1%). Over half of the participants visited church sometimes (58.9%) and most of them reported having children or friends (73.1% and 86.4%, respectively). According to the EURO-D scale, 160 (45.8%) had case-level depressive symptoms (EURO-D score ≥ 4). Those with depression had significantly lower education levels and were less likely to attend church. They also presented a higher number of stressful life events than those without depressive symptoms. The genotype frequencies did not deviate from the HWE (*P* > 0.05) and reflected frequencies previously observed in white European populations^14,29,44^. The frequency of the *5-HTTLPR* genotypes and alleles in elderly population did not differ significantly according to EURO-D scale score.

**Table 1:** The basic characteristics of study population.

Characteristic	Whole sample (n = 353)	EURO-D scale (score)		P value
		< 4 (n = 189)	≥ 4 (n = 160)	
Age	73.06	73.00	74.49	0.220
Median (min, max)	(65.00, 99.93)	(65.01, 99.93)	(65.00, 96.99)	
Sex (female), n (%)	243 (67.1)	124 (65.6)	117 (73.1)	0.130
	110 (30.4)	65 (34.4)	43 (26.9)	
Marital status, n (%)				
Single	25 (7.1)	13 (6.9)	12 (7.5)	0.200
Married or stable partners	152 (43.4)	86 (45.5)	65 (40.9)	
Divorced	29 (8.3)	20 (10.6)	9 (5.7)	
Widowed	144 (41.1)	70 (37.0)	73 (45.9)	
No information	3	–	1	
Education, n (%)				
Primary school	122 (34.8)	48 (25.4)	73 (45.6)	**< 0.001**
Secondary school	101 (28.8)	61 (32.3)	39 (24.4)	
Higher and university education	128 (36.5)	80 (42.3)	48 (30.0)	
No information	2	–	–	
Church attendance, n (%)				
No	48 (13.7)	18 (9.5)	29 (18.2)	**0.042**
Sometimes	206 (58.9)	113 (59.8)	92 (57.9)	
Regular	96 (27.4)	58 (30.7)	38 (23.9)	
No information	3	–	1	
Social activity, n (%)				
No	280 (79.3)	142 (75.1)	136 (85.0)	0.194
Sometimes	40 (11.3)	25 (13.2)	15 (9.4)	
Regular	27 (7.6)	19 (10.1)	8 (5.0)	
No information	6	3	1	
Having friends, n (%)				
No	93 (26.9)	49 (26.5)	43 (27.0)	0.907
Yes	253 (73.1)	136 (73.5)	116 (73.0)	
No information	7	4	1	
Meetings with friends, n (%)				
Never	33 (10.9)	15 (9.1)	18 (13.0)	0.694
At least once per month	60 (19.7)	34 (20.7)	26 (18.8)	
At least once a week/2–3 times a week	115 (37.8)	65 (39.6)	50 (36.2)	
Every day	96 (31.6)	50 (30.5)	44 (31.9)	
No information	49	25	22	
Having children, n (%)				
No	37 (10.5)	17 (9.3)	20 (12.7)	0.327
Yes	305 (86.4)	165 (90.7)	138 (87.3)	
No information	11	7	2	
Meetings with children, n (%)				
Never	3 (0.9)	1 (0.5)	2 (1.3)	0.367
At least once per month	75 (21.6)	25 (18.5)	39 (24.8)	
At least once a week/2–3 times a week	128 (36.8)	75 (39.7)	52 (33.1)	
Every day	142 (40.8)	78 (41.3)	60 (40.8)	
No information	5	–	3	
Lifetime stressful events (LSE) scale score				
Median (min, max)	3 (0, 11)	3 (0, 9)	3 (0, 11)	**0.006**
No information	51	29	20	
Current stressful events (CSE) scale score				
Median (min, max)	0 (0, 6)	0 (0, 4)	0 (0, 6)	**< 0.001**
No information	50	26	22	
Genotypes of *5-HTTLPR*, n (%)				
l/l	120 (40.3)	66 (41.0)	53 (39.3)	0.436
l/s	150 (50.3)	83 (51.6)	66 (48.9)	
s/s	28 (9.4)	12 (7.5)	16 (11.9)	
s allele frequency	206 (34.6)	107 (33.2)	98 (36.3)	0.435
l allele frequency	390 (65.4)	215 (66.8)	172 (63.7)	
No information	55	28	25	
Genotypes of *5-HTTLPR*, n (%)				
l/l	120 (40.3)	66 (41.0)	53 (39.3)	0.762
l/s or s/s	178 (59.7)	95 (59.0)	82 (60.7)	
No information	55	28	25	

Significant values are in bold.

*5-HTTLPR* polymorphism in the promoter region of the serotonin transporter (5-HTT) encoding gene (*SLC6A4)*, *EURO-D* depression symptom scale.

After adjustment for age, univariate logistic regression analysis revealed that LSE (*P* = 0.013), CSE (*P* < 0.001), and primary school (vs. higher) education (*P* < 0.001) were significantly associated with higher odds of depression, while regular church attendance (*P* = 0.017) and social activity (borderline significant) were associated with decreased odds (Table 2). Including the significant variables in the multivariate logistics regression model, we found that CSE (aOR 2.292, 95% CI 1.569–3.349, *P* < 0.001) and primary school education (aOR 3.392, 95% CI 1.824–6.306, *P* < 0.001) retained independent significance.

**Table 2:** Univariate and multivariate logistic regression analysis to predict depression*.

Variable	Univariate logistic regression model		Multivariate logistic regression model	
	aOR (95% CI)	P value	aOR (95% CI)	P value
Sex				
Female vs male	1.427 (0.899–2.265)	0.131	–	
LSE	1.170 (1.034–1.324)	**0.013**	1.142 (0.999–1.305)	0.052
CSE	2.229 (1.574–3.156)	**< 0.001**	2.292 (1.569- 3.349)	**< 0.001**
Marital status				
Married or stable partners	1			
Single	1.196 (0.511–2.799)	0.680	–	
Divorced	0.602 (0.257–1.410)	0.242	–	
Widowed	1.312 (0.818–2.104)	0.260	–	
Education				
Higher and university education	1		1	
Primary school	2.476 (1.476–4.156)	**< 0.001**	3.392 (1.824–6.306)	**< 0.001**
Secondary school	1.072 (0.626–1.838)	0.799	1.576 (0.806–3.081)	0.184
Church attendance				
No	1			
Sometimes	0.533 (0.276–1.028)	0.060	–	
Regular	0.416 (0.203–0.854)	**0.017**	–	
Social activity				
No	1			
Sometimes	0.456 (0.192–1.080)	0.221	–	
Regular	0.651 (0.328–1.294)	0.074	–	
Having friends				
Yes vs no	1.027 (0.632–1.671)	0.913	–	
Meetings with friends				
Never	1			
At least once a month	0.663 (0.281–1.565)	0.348	–	
At least once a week/2–3 times a week	0.679 (0.309–1.492)	0.335	–	
Every day	0.764 (0.343–1.704)	0.511	–	
Having children				
Yes vs no	0.748 (0.375–1.494)	0.411	–	
Meetings with children				
Never	1			
At least once a month	0.574 (0.050–6.657)	0.657	–	
At least once a week/2–3 times a week	0.352 (0.031–4.006)	0.400	–	
Every day	0.408 (0.036–4.635)	0.470	–	
Genotypes of *5-HTTLPR*				
l/l	1			
l/s	0.989 (0.608–1.609)	0.965	–	
s/s	1.748 (0.757–4.037)	0.191	–	
Genotypes of *5-HTTLPR*				
l/l	1			
l/s or s/s	1.082 (0.677–1.728)	0.742	–	

Significant values are in bold.

* Having high levels of depression symptomatology according to EURO-D (score ≥ 4); *LSE* lifetime stressful events (at least one recent stressful event during lifetime), *CSE* current stressful events (at least one stressful event that happened in the last 6 months), *5-HTTLPR* polymorphism in the promoter region of the serotonin transporter (5-HTT) encoding gene (*SLC6A4*), *aOR* odds ratio adjusted for age, *CI* confidence interval.

In univariate age-adjusted logistic regression analyses (Table 3), a *5-HTTLPR* × LSE interaction effect on depression was observed in that highest odds of depression were found in participants with both high stress and s/s genotype for LSE; for CSE, this highest-risk group was observed both for the s/s genotype and when analysed for presence of the *s* allele (i.e., s/s and s/l combined) with a dose–response pattern from the low stress × l/l combination to the high stress × s/s combination.

**Table 3:** Univariate logistic regression analysis of the *5-HTTLPR* and traumatic stressful events interaction to predict depression*.

Variable		Univariate logistic regression	
Genotypes of *5-HTTLPR* × LSE^a^		aOR (95% CI)	P value
l/l (n = 37)		1	
l/s (n = 42)	Low/medium stress	1.052 (0.416–2.661)	0.915
s/s (n = 10)		1.354 (0.320–5.734)	0.681
l/l (n = 56)		1.902 (0.805–4.492)	0.143
l/s (n = 92)	High stress	1.728 (0.782–3.822)	0.177
s/s (n = 15)		5.306 (1.396–20.177)	**0.014**
Genotypes of *5-HTTLPR* × LSE^a^			
l/l (n = 37)	Low/medium stress	1	0.821
l/s or s/s (n = 52)		1.107 (0.458–2.673)	
l/l (n = 56)	High stress	1.906 (0.807–5.500)	0.141
l/s or s/s (n = 107)		1.997 (0.917–4.349)	0.081
Genotypes of *5-HTTLPR* × CSE^b^			
l/l (n = 71)		1	
l/s (n = 94)	Not exposed to stress	0.883 (0.458–1.704)	0.711
s/s (n = 19)		2.309 (0.815–6.538)	0.115
l/l (n = 24)		4.034 (1.498–10.864)	**0.006**
l/s (n = 39)	Exposed to stress	6.574 (2.669–16.191)	**< 0.001**
s/s (n = 6)		10.675 (1.169–97.488)	**0.036**
Genotypes of *5-HTTLPR* × CSE^b^			
l/l (n = 71)	Not exposed to stress	1	
l/s or s/s (n = 113)		1.045 (0.559–1.954)	0.891
l/l (n = 24)	Exposed to stress	3.985 (1.482–10.715)	**0.006**
l/s or s/s (n = 45)		6.899 (2.903–16.393)	**< 0.001**

Significant values are in bold.

* Having high levels of depression symptomatology according to EURO-D (score ≥ 4); *5-HTTLPR* polymorphism in the promoter region of the serotonin transporter (5-HTT) encoding gene (*SLC6A4*), *aOR* odds ratio adjusted for age, *CI* confidence interval.

^a^The two categories of lifetime stressful events (LSE) were included in analysis: low/medium stress—0–2, high stress—≥ 3 number of negative life events.

^b^The two categories of current stressful events (CSE) were included in analysis: not exposed to stress—0, exposed to stress—≥ 1 number of negative life events.

## Discussion

This study investigated the association between *5-HTTLPR* polymorphism, its interaction with stressful life events, and risk of late-life depressive symptoms in a sample of older people from a Lithuanian catchment. Consistent with previous studies^33–35^, we found no significant direct association between the *5-HTTLPR s* allele and late-life depressive symptoms. On the other hand, our data replicated the results of two meta-analyses^34,35^, in the strong associations found between stressful life events and depressive symptoms, strongest for more recent stressful events.

One of the most recent meta-analyses of this polymorphism^45^ concluded that *5-HTTLPR* and stress interaction is a dynamic process, producing different effects at different time points. Data show that the effect of the *5-HTTLPR* x stress interaction emerged only in the case of chronic stress, i.e. the risk of depression by *5-HTTLPR* genotypes emerges with time and the difference is seen only following chronic stress. It is widely acknowledged that chronic stress, more than acute stress, is a key factor for the onset of depression^46^. However, our *5-HTTLPR* and stressful events interaction analyses show that both lifetime or current stressful life events remain important factors for the onset of depression, and that *5-HTTLPR* genotype potentially modulates the development of depression. Specifically, analyses show a dose-dependent effect of number of stressful events experienced by elders and the role of *HTTLPR* genotype. These results support previous conclusions^14,47^ that *5-HTTLPR* genotype modifies the strength of association between stressful events and depressive symptoms. In our sample, the odds of depression were increased in *s* homozygous participants who experienced high lifetime stress compared to the *l* homozygotes with low or medium stress. Our results also confirm prior findings^14^ that increasing numbers of *5-HTTLPR* s alleles confer higher vulnerability to depressive symptoms associated with recent life events. These findings support the *5-HTTLPR* × stress interaction hypothesis and suggest that the effect of the interaction on depression risk is dose-dependent on the number of stressful events.

The prevalence of depressive symptoms varies according to geographical region, with rates being lower in northern Europe and higher in southern and eastern Europe countries^2^. The prevalence of late-life depression displays considerably higher rates in the oldest compared with the youngest age group. Studies that have used the EURO-D scale to assess depressive symptoms in European countries have reported prevalence rates, between 17.8 and 38.3% in older population aged 50 and over^2^ and 15.8–41.4% in people aged 65 and over^48,49^. The prevalence of depressive symptoms (EURO-D ≥ 4) in our sample (45.8%) also support previous findings.

Considering other findings, depressive symptoms were more frequent in older people with lower educational attainment. This is consistent with conclusions from other studies that higher education is associated with decreased risk of late-life depression^2,50,51^. Practice of religion may also serve as a protective factor against depression in older people^2,52^ and our study replicated prior findings showing a strong association between church attendance and reduced likelihood of depression^53,54^. Previous studies have found that intergenerational contact frequency is independently and inversely associated with depressive symptoms in the elderly^55,56^. Our findings show that the odds of depressive symptoms decreased with increasing frequency of meetings with children or friends; however, the associations failed to reach statistical significance.

This study has several limitations. First, depressive symptoms were ascertained as an outcome according to a cut-off score on a screening instrument, albeit one that has been widely used and validated in previous studies, and findings cannot necessarily be applied to diagnostic categories. Second, we did not seek to ascertain or analyse stress-related factors experienced in childhood and adulthood separately. Third, potentially important factor such as use of medication was not included in the analysis. Despite these limitations, the strengths of our study are that we used EURO-D scale which was originally developed to compare symptoms of LLD across European countries^41^ and has been validated and used in many epidemiological studies. To our knowledge, this is the first study investigating the impact of current and lifetime stressful life events on the occurrence of depressive symptoms in an elderly Lithuanian population. This population-based study is homogeneous by genetic ancestry and adds further informative evidence not only about the impact of stress-related life events as risk factors for depression onset, but also presents effect of sociodemographic factors on the development of depression in the elderly. In addition, the evaluation of the *5-HTTLPR* and traumatic stressful events interaction demonstrated the significance of genetic variation in phenotype as LLD.

In conclusion, although no significant association was found for elderly carrying the short allele of the *5-HTTLPR* and being particularly susceptible to depression, our findings demonstrated that lifetime or current stressful life events and their interaction with the *5-HTTLPR* genotype significantly increased odds of late-life depression in elderly population.

## Methods

### Sample collection

For the Kaunas Healthy Ageing Study, a sample of 400 participants of both sexes over 65 years old was randomly selected from the list of Kaunas city inhabitants between 2006 and 2009 by the Residents’ Register Service of Lithuania. From this list, 197 persons agreed to participate in the Study. Because of inclusion difficulties (not possible to find at home after repeated visits, not having a telephone, refusing to participate), other participants were included using a snowball sampling method and recommendation from study subjects. In total, 353 persons participated in the study. Of these, 298 agreed to provide blood samples for genetic study. All the participants were Caucasian and Lithuanian.

The study was approved by Kaunas Regional Biomedical Research Ethics Committee (Lithuania) (permission number BE-2-49/2008). In accordance with the Declaration of Helsinki, written informed consent was obtained from each participant and aims of the study had been fully explained. All clinical and laboratory data was coded and entered into a secure computer database only accessible to the research study team.

### Mental health and psychological status assessment

A questionnaire, which covered health, psychological, social support, and economic variables, was administered by trained interviewers in the respondents’ homes. Depressive symptoms were assessed using the EURO-D scale^41^, which consists of 12 items: depression affect, pessimism, wishing to die, guilt, sleep, interest, irritability, appetite, fatigue, concentration, enjoyment, and tearfulness during the last month. Each item receives a binary present/absent score, creating an ordinal scale with a maximum score of 12. Scores of 4 or more were classified as case-level depressive symptoms, as previously reported to indicate the presence of major depression^41^. The List of Threatening Events Questionnaire (LTE-Q)^42^ composed of 12 items was used to identify traumatic stressful life events that happened in the last 6 months (current stressful events, CSE) and during lifetime (lifetime stressful events, LSE) related to relationship breakdown, financial difficulties, illnesses/injuries and death of family or friends. In addition, all respondents were asked questions related to physical and sexual abuse, violence at home and at work, expulsion from school and homelessness. For each event, binary present/absent codes were allocated and the sum of scores was calculated as the number of stressful events and used for statistical analysis. For the *5-HTTLPR* × traumatic stressful events (LSE or CSE) interaction analysis to predict depression, the LSE scores were grouped into two categories (low/medium stress—0–2, high stress—≥ 3), and the CSE scores were divided into two categories (not exposed to stress—0, exposed to stress—≥ 1) consistent with previous studies^14,35^.

### *5-HTTPLR* genotyping

Genomic DNA was extracted from venous blood (3 ml) using Sorpoclean Genomic DNA Extraction Module kit (Sorpo Diagnostics, Vilnius, Lithuania) according to the manufacturer’s instructions. The *5-HTT* regulatory gene region was amplified using a polymerase chain reaction (PCR) with oligonucleotide primers (Matabion, Germany): forward: 5′-GGCGTTGCCGCTCTGAATGC-3′; reverse: 5′-GAGGGACTGAGCTGGACAACCAC-3′. PCR was performed in a 25 ml volume containing approximately 50 ng of genomic template, 0.4 µmol/l of each primer, 200 µmol/l of each deoxynucleotide triphosphate (dNTP), 2.5 units of *Taq* polymerase, 1.5 mmol/l MgCl_2_, 5% dimethyl sulfoxide (DMSO), 1× PCR Buffer (“Thermo Fisher Scientific”, Lithuania). Cycling conditions were preceded by a denaturing step at 95 °C for 10 min, followed by 35 cycles of denaturation at 95 °C for 30 s, annealing at 63 °C for 30 s, and synthesis at 72 °C for 80 s. The reaction was ended by incubation at 72 °C for 7 min. PCR products were separated by electrophoresis (100 V for 2.5 h; 2.5% agarose gel) and visualized by ethidium bromide-stained agarose gel under ultraviolet light using a video documentation system, the BioDocAnalyse 2.0 (Biometra, Göttingen, Germany). Sequined DNA standards were used to identify the 484/528 bp corresponding short (s) and long (l) fragments of *5-HTTLPR*.

PCR data were validated using a TaqMan 5’nuclease assay modified from that originally described by Ref.^43^. The 25-μl PCR reactions contained 100 nM of each forward and reverse primers (GCAACCTCCCAGCAACTCCCTGTA and GAGGTGCAGGGGGATGCTGGAA), 120 nM of an l allele specific FAM-labeled probe, and 60 nM of a VIC-labeled internal control probe whose target is present in the PCR amplicon for both l alleles and s alleles (6FAM-TGCAGCCCCCCCAGCATCTCCCMGB and VIC-TCCCCCCCTTCACCCCTCGCGGCATCC-MGB), (Applied Biosystems, USA); 4% DMSO (“Thermo Fisher Scientific”, Lithuania); 1× TaqMan Universal master mix (Applied Biosystems, USA); and 20 ng genomic DNA. Samples were heated to 95 °C for 10 min to activate *Taq* DNA polymerase, followed by 40 thermal cycles of 95 °C for 15 s, followed by 62.5 °C for 90 s. The number of alleles for each subject was identified by examination of scatter plots of end point FAM versus VIC fluorescence levels captured using an ABI 7900HT Sequence Detection System with SDS v 2.1 (Applied Biosystems, Foster City, CA, USA). There was no discrepancy in 5-*HTTLPR* genotype between the 5′nuclease TaqMan and conventional agarose gel-based PCR fragment length assays.

### Statistical analysis

Quantitative variables were described as median (minimum, maximum), because variable distributions did not satisfy the normality assumption (Kolmogorov–Smirnov or Shapiro–Wilk tests). Nonparametric Mann–Whitney U tests were used to determine differences in the distributions of continuous variables between two independent samples, and Kruskal–Wallis for three independent samples. Categorical variables were described using frequencies and percentages. The χ^2^ test was used to determine differences between the categorical variables and for the assessment of the Hardy–Weinberg equilibrium (HWE) for the distribution of genotypes. Univariate and multivariate binary logistic regression analyses were applied to evaluate the associations between adverse life events, *5-HTTLPR* genotype and the odds ratio of depression according to EURO-D scale (score). Univariate binary logistic regression analysis was applied to evaluate the *5-HTTLPR* × Traumatic stressful events (LSF or CSF) interaction to predict depression. Adjusted odds ratio (aOR) with 95% confidence interval was calculated. Statistical analysis was performed using the statistical software package IBM SPSS Statistics version 27 for Windows. *P* value < 0.05 was considered statistically significant.

### Ethics statement

The study was approved by Kaunas Regional Biomedical Research Ethics Committee (Lithuania) (permission number BE-2-49/2008). In accordance with the Declaration of Helsinki, written informed consent was obtained from each participant and aims of the study had been fully explained. All clinical and laboratory data was coded and entered into a secure computer database only accessible to the research study team.

## Data Availability

The data generated and analysed during the study are available from the corresponding author upon reasonable request. Data will be stripped from all information allowing identification of study participants.

## References

[1] Basta MM (2021). Frequency and risk factors associated with depression in elderly visiting Primary Health Care (PHC) settings: Findings from the Cretan Aging Cohort. J. Affect. Dis. Rep..

[2] Costa EC (2007). Prevalence of depressive symptoms and syndromes in later life in ten European countries: The SHARE study. Br. J. Psych..

[3] Van de Velde S, Bracke P, Levecque K (2010). Gender differences in depression in 23 European countries. Cross-national variation in the gender gap in depression. Soc. Sci. Med..

[4] Hansen T, Slagsvold B (2017). The East-West divide in late-life depression in Europe: Results from the Generations and Gender Survey. Scand. Psychol..

[5] Cheruvu VK, Chiyaka ET (2019). Prevalence of depression symptoms among older adults who reported medical cost as a barrier to seeking health care: Findings from a nationally representative sample. BMC Geriat..

[6] Blazer DG (2003). Depression in late life: review and commentary. J. Gerontol. A. Biol. Sci. Med. Sci..

[7] Van Damme A, Declercq T, Lemey L, Tandt H, Petrovic M (2018). Late-life depression: Issues for the general practitioner. Int. J. Gen. Med..

[8] Linnemann C, Lang UE (2020). Pathways Connecting late-life depression and dementia. Front. Pharmacol..

[9] Kessler RC (2010). Age differences in major depression: Results from the national comorbidity survey replication (NCS-R). Psychol. Med..

[10] Gertner AK, Domino ME, Dow WH (2017). Risk factors for late-life depression and correlates of antidepressant use in Costa Rica: Results from a nationally representative longitudinal survey of older adults. J. Affect. Disord..

[11] Fiske A, Wetherell JL, Gatz M (2009). Depression in older adults. Annu. Rev. Clin. Psychol..

[12] Prince MJ, Harwood RH, Thomas A, Mann AH (1998). A prospective population-based cohort study of the effects of disablement and social milieu on the onset and maintenance of late-life depression. The Gospel Oak Project VII. Psych. Med..

[13] Bøe T, Balaj M, Eikemo TA, McNamara CL, Solheim EF (2017). Financial difficulties in childhood and adult depression in Europe. Eur. J. Public Health..

[14] Juhasz G (2015). Variability in the effect of 5-HTTLPR on depression in a large european population: The role of age, symptom profile, type and intensity of life stressors. PLoS ONE.

[15] Köhler CA (2018). Mapping risk factors for depression across the lifespan: An umbrella review of evidence from meta-analyses and Mendelian randomization studies. J. Psychiatr. Res..

[16] Heim C, Plotsky PM, Nemeroff CB (2004). Importance of studying the contributions of early adverse experience to neurobiological findings in depression. Neuropsychopharmacology.

[17] Houwing DJ, Buwalda B, van der Zee EA, de Boer SF, Olivier JDA (2017). The serotonin transporter and early life stress: Translational perspectives. Front. Cell Neurosci..

[18] Ryan J, Ancelin ML (2019). Genetic and epigenetic regulation of the serotonin transporter gene in late-life depression. J. Geriatr. Psychiatry Neurol..

[19] Andrews PW, Bharwani A, Lee KR, Fox M, Thomson JA (2015). Is serotonin an upper or a downer? The evolution of the serotonergic system and its role in depression and the antidepressant response. Neurosci. Biobehav. Rev..

[20] Lam D, Ancelin ML, Ritchie K (2018). Genotype-dependent associations between serotonin transporter gene (*SLC6A4*) DNA methylation and late-life depression. BMC Psychiatry.

[21] Heils A (1996). Allelic variation of human serotonin transporter gene expression. J. Neurochem..

[22] Lesch KP (1996). Association of anxiety-related traits with a polymorphism in the serotonin transporter gene regulatory region. Science.

[23] Nakamura M, Ueno S, Sano A, Tanable H (2000). The human serotonin transporter gene-linked polymorphism (5-HTTLPR) shows ten novel allelic variants. Mol. Psychiatry..

[24] O’Hara R, Hallmayer JF (2007). Serotonin transporter polymorphism and stress: A view across the lifespan. Curr. Psychiatry Rep..

[25] Caspi A (2003). Influence of life stress on depression: Moderation by a polymorphism in the 5-HTT gene. Science.

[26] Kaufman J (2004). Social supports and serotonin transporter gene moderate depression in maltreated children. Proc. Nat. Acad. Sci. U. S. A..

[27] Kim JM (2007). Interactions between life stressors and susceptibility genes (5-HTTLPR and BDNF) on depression in Korean elders. Biol. Psychiatry..

[28] Xie P (2009). Interactive effect of stressful life events and the serotonin transporter 5-HTTLPR genotype on posttraumatic stress disorder diagnosis in 2 independent populations. Arch. Gen. Psychiatry..

[29] Davin A (2016). Correction: Influence of serotonin transporter gene polymorphisms and adverse life events on depression symptoms in the elderly: A population-based study. PLoS ONE.

[30] Karg K, Burmeister M, Shedden K, Sen S (2011). The serotonin transporter promoter variant (5-HTTLPR), stress, and depression meta-analysis revisited: evidence of genetic moderation. Arch. Gen. Psychiatry..

[31] Sharpley CF, Palanisamy SK, Glyde NS, Dillingham PW, Agnew LL (2014). An update on the interaction between the serotonin transporter promoter variant (5-HTTLPR), stress and depression, plus an exploration of non-confirming findings. Behav. Brain Res..

[32] Power T (2010). 5-HTTLPR genotype, stressful life-events and late-life depression: No evidence of interaction in a French population. Neurobiol. Aging..

[33] Munafo MR, Durrant C, Lewis G, Flint J (2008). Gene × environment interactions at the serotonin transporter locus. Biol. Psychiatry..

[34] Risch N (2009). Interaction between the serotonin transporter gene (5-HTTLPR), stressful life events, and risk of depression: A meta-analysis. JAMA.

[35] Culverhouse RC (2018). Collaborative meta-analysis finds no evidence of a strong interaction between stress and 5-HTTLPR genotype contributing to the development of depression. Mol. Psychiatry..

[36] Grabe H (2011). Update on the 2005 paper: Moderation of mental and physical distress by polymorphisms in the *5-HT* transporter gene by interacting with social stressors and chronic disease burden. Mol. Psychiatry..

[37] Duncan LE, Keller MC (2011). A critical review of the first 10 years of candidate gene-by-environment interaction research in psychiatry. Am. J. Psychiatry..

[38] Weeland J, Overbeek G, de Castro BO, Matthys W (2015). Underlying mechanisms of gene-environment interactions in externalizing behavior: A systematic review and search for theoretical mechanisms. Clin. Child. Fam. Psychol. Rev..

[39] Woodward AA, Urbanowicz RJ, Naj AC, Moore JH (2022). Genetic heterogeneity: Challenges, impacts, and methods through an associative lens. Genet. Epidemiol..

[40] Enoch MA, Shen PH, Xu K, Hodgkinson C, Goldman D (2006). Using ancestry-informative markers to define populations and detect population stratification. J. Psychol. Pharm..

[41] Prince MJ (1999). Development of the EURO-D scale-a European, Union initiative to compare symptoms of depression in 14 European centres. Br. J. Psychiatry..

[42] Brugha T, Bebbington P, Tennant C, Hurry J (1985). The list of threatening experiences: A subset of 12 life event categories with considerable long-term contextual threat. Psychol. Med..

[43] Covault J (2007). Interactive effects of the serotonin transporter 5-HTTLPR polymorphism and stressful life events on college student drinking and drug use. Biol. Psychiatry..

[44] Ancelin ML (2017). Heterogeneity in HPA axis dysregulation and serotonergic vulnerability to depression. Psychoneuroendocrinology.

[45] Colli CD (2022). Time moderates the interplay between 5-HTTLPR and stress on depression risk: Gene x environment interaction as a dynamic process. Transl. Psychiatry..

[46] McGonagle KA, Kessler RC (1990). Chronic stress, acute stress, and depressive symptoms. Am. J. Commun. Psychiatry..

[47] Rozenblat V (2017). A systematic review and secondary data analysis of the interactions between the serotonin transporter 5-HTTLPR polymorphism and environmental and psychological factors in eating disorders. J. Psychiatr. Res..

[48] Conde-Sala JL, Portellano-Ortiz C, Calvó-Perxas L, Garre-Olmo J (2017). Quality of life in people aged 65+ in Europe: associated factors and models of social welfare-analysis of data from the SHARE project (Wave 5). Qual. Life Res..

[49] Belvederi MM, Amore M, Respino M (2020). The symptom network structure of depressive symptoms in late-life: Results from a European population study. Mol. Psychiatry..

[50] Chang-Quan H, Zheng-Rong W, Yong-Hong L, Yi-Zhou X, Qing-Xiu L (2010). Education and risk for late life depression: A meta-analysis of published literature. Int. J. Psychiatry..

[51] Kleisiaris C (2013). The prevalence of depression symptoms in an elderly population and their relation to life situations in home care. Health Sci. J..

[52] Hayward RD, Owen AD, Koenig HG, Steffens DC, Payne ME (2012). Longitudinal relationships of religion with posttreatment depression severity in older psychiatric patients: Evidence of direct and indirect effects. Depress. Res. Treat..

[53] Ronneberg CR, Miller EA, Dugan E, Porell F (2016). The protective effects of religiosity on depression: A 2-year prospective study. Geront..

[54] Van Herreweghe L, Van Lancker W (2019). Is religiousness really helpful to reduce depression symptoms at old age? A longitudinal study. PLoS ONE.

[55] Tosi M, Grundy E (2019). Intergenerational contacts and depressive symptoms among older parents in Eastern Europe. Aging Ment. Health..

[56] Xie Y (2020). Dose–response relationship between intergenerational contact frequency and depressive symptoms amongst elderly Chinese parents: A cross-sectional study. BMC Geriat..

